# Magnetoelectrics for Implantable Bioelectronics: Progress to Date

**DOI:** 10.1021/acs.accounts.4c00307

**Published:** 2024-10-04

**Authors:** Fatima Alrashdan, Kaiyuan Yang, Jacob T. Robinson

**Affiliations:** † Department of Electrical and Computer Engineering, Rice University, 6100 Main St, Houston, Texas 77005, United States; ‡ Department of Bioengineering, Rice University, 6100 Main St, Houston, Texas 77005, United States; § Applied Physics Program, Rice University, 6100 Main St, Houston, Texas 77005, United States; ∥ Department of Neuroscience, Baylor College of Medicine, 1 Baylor Plaza, Houston, Texas 77030, United States

## Abstract

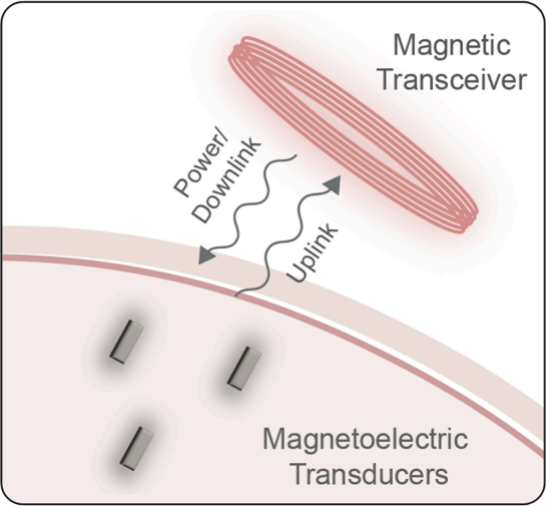

The coupling of magnetic and electric properties manifested in magnetoelectric (ME) materials has unlocked numerous possibilities for advancing technologies like energy harvesting, memory devices, and medical technologies. Due to this unique coupling, the magnetic properties of these materials can be tuned by an electric field; conversely, their electric polarization can be manipulated through a magnetic field.

Over the past seven years, our lab work has focused on leveraging these materials to engineer implantable bioelectronics for various neuromodulation applications. One of the main challenges for bioelectronics is to design miniaturized solutions that can be delivered with minimally invasive procedures and yet can receive sufficient power to directly stimulate tissue or power electronics to perform functions like communication and sensing.

Magnetoelectric coupling in ME materials is strongest when the driving field matches a mechanical resonant mode. However, miniaturized ME transducers typically have resonance frequencies >100 kHz, which is too high for direct neuromodulation as neurons only respond to low frequencies (typically <1 kHz). We discuss two approaches that have been proposed to overcome this frequency mismatch: operating off-resonance and rectification. The off-resonance approach is most common for magnetoelectric nanoparticles (MENPs) that typically have resonance frequencies in the gigahertz range. In vivo experiments on rat models have shown that MENPs could induce changes in neural activity upon excitation with <200 Hz magnetic fields. However, the neural response has latencies of several seconds due to the weak coupling in the off-resonance regime.

To stimulate neural responses with millisecond precision, we developed methods to rectify the ME response so that we could drive the materials at their resonant frequency but still produce the slowly varying voltages needed for direct neural stimulation. The first version of the stimulator combined a ME transducer and analog electronics for rectification. To create even smaller solutions, we introduced the first magnetoelectric metamaterial (MNM) that exhibits self-rectification. Both designs have effectively induced neural modulation in rat models with less than 5 ms latency.

Based on our experience with in vivo testing of the rectified ME stimulators, we found it challenging to deliver the precisely controlled therapy required for clinical applications, given the ME transducer’s sensitivity to the external transmitter alignment. To overcome this challenge, we developed the ME-BIT (MagnetoElectric BioImplanT), a digitally programmable stimulator that receives wireless power and data through the ME link.

We further expanded the utility of this technology to neuromodulation applications that require high stimulation thresholds by introducing the DOT (Digitally programmable Overbrain Therapeutic). The DOT has voltage compliance up to 14.5 V. We have demonstrated the efficacy of these designs through various in vivo studies for applications like peripheral nerve stimulation and epidural cortical stimulation.

To further improve these systems to be adaptive and enable a network of coordinated devices, we developed a bidirectional communication system to transmit data to and from the implant. To enable even greater miniaturization, we developed a way to use the same ME transducer for wireless power and data communication by developing the first ME backscatter communication protocol.

## Key References

- SingerA.; DuttaS.; LewisE.; ChenZ.; ChenJ. C.; VermaN.; AvantsB.; FeldmanA. K.; O’MalleyJ.; BeierleinM.; KemereC.; RobinsonJ. T.Magnetoelectric Materials for Miniature, Wireless Neural Stimulation at Therapeutic Frequencies. Neuron2020, 107, 631–643.e532516574 10.1016/j.neuron.2020.05.019PMC7818389.^1^ First in vivo demonstration of mm-sized magnetoelectric-powered neural stimulators operating at therapeutic frequencies near 130 Hz.
- ChenJ. C.; KanP.; YuZ.; AlrashdanF.; GarciaR.; SingerA.; LaiC. S. E.; AvantsB.; CrosbyS.; LiZ.; WangB.; FelicellaM. M.; RobledoA.; PeterchevA. V.; GoetzS. M.; HartgerinkJ. D.; ShethS. A.; YangK.; RobinsonJ. T.A Wireless Millimetric Magnetoelectric Implant for the Endovascular Stimulation of Peripheral Nerves. Nat. Biomed. Eng.2022, 6, 706–71635361934 10.1038/s41551-022-00873-7PMC9213237.^2^ First digitally programmable millimeter-sized magnetoelectric-powered neural stimulator used in vivo.
- YuZ.; AlrashdanF. T.; WangW.; ParkerM.; ChenX.; ChenF. Y.; WoodsJ.; ChenZ.; RobinsonJ. T.; YangK.Magnetoelectric Backscatter Communication for Millimeter-Sized Wireless Biomedical Implants. In Proceedings of the 28th Annual International Conference on Mobile Computing And Networking; MobiCom ’22; Association for Computing Machinery: New York, NY, USA, 2022; pp 432–445,10.1145/3495243.3560541..^3^ First near-zero power magnetoelectric backscatter communication system for mm-sized bioimplants.
- ChenJ. C.; BhaveG.; AlrashdanF.; DhuliyawallaA.; HoganK. J.; MikosA. G.; RobinsonJ. T.Self-Rectifying Magnetoelectric Metamaterials for Remote Neural Stimulation and Motor Function Restoration. Nat. Mater.2024, 23, 13937814117 10.1038/s41563-023-01680-4PMC10972531.^4^ First self-rectifying magnetoelectric metamaterial. Used here for precisely timed neural stimulation with less than 5 ms latencies.
- WoodsJ. E.; SingerA. L.; AlrashdanF.; TanW.; TanC.; ShethS. A.; ShethS. A.; RobinsonJ. T.Miniature Battery-Free Epidural Cortical Stimulators. Science Advances2024, 10, eadn085838608028 10.1126/sciadv.adn0858PMC11014439.^5^ First demonstration of a magnetoelectric-powered stimulator in humans.

## History of Magnetoelectric Materials

The use of magnetic and electric materials has fueled two centuries of advances in communications, energy production, and medicine, but only in the last 30 years have materials that exhibit both electric and magnetic properties begun to enter the technology sector. Before 1894, magnetic and electric properties were considered mutually exclusive.^6^ Electric materials require vacant d-orbitals to exhibit spontaneous polarization, whereas magnetic materials depend on unpaired electrons in partially filled d-orbitals to generate magnetic moments.^7^ Therefore, the coexistence of magnetic and electric properties in a single material was perceived as contradictory. Pierre Curie’s work on crystal symmetry in 1894 challenged this notion, suggesting that electric and magnetic orders could coexist in the same material.^8^ It would take over half a century before Astrov’s experimental work confirmed Curie’s prediction by demonstrating that materials like single crystal Cr_2_O_3_ could have their magnetic moment manipulated by an electric field at 0 C.^9^ This breakthrough led to the emergence of a new class of materials known as magnetoelectric (ME) materials. The electric polarization of such materials can be controlled through a magnetic field, also known as the direct magnetoelectric effect. Similarly, the magnetic moment can be manipulated through an applied electric field, also known as the converse magnetoelectric effect. The strength of this coupling is quantified by the magnetoelectric coupling coefficient defined as^10^
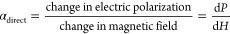

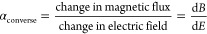


**Figure 1:**
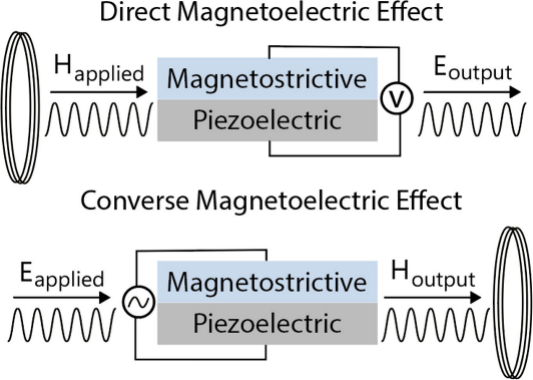
Illustration of direct and converse magnetoelectric effects in composite magnetoelectric materials.

However, the initial excitement for this discovery waned as it became clear that natural magnetoelectric compounds either exhibited weak coupling or only showed coupling at low temperatures, limiting any practical application. To overcome these limitations, Van Suchtelen introduced the concept of composite materials. To enhance the magnetoelectric coupling, Suchtelen suggested combining two materials: a magnetostrictive material with magnetic and elastic orders and a piezoelectric material with elastic and electric orders.^11^ Hence, the magnetoelectric coupling (Figure1) results from indirect energy transduction through elastic energy (magnetic ↔ elastic ↔ electric). This two-phase material exhibits a strain-mediated magnetoelectric coupling, offering a promising solution to the limitations encountered with natural magnetoelectric compounds. Soon after, composite magnetoelectric materials, consisting of spinel cobalt ferrite (magnetostrictive) and perovskite barium titanate (piezoelectric), were successfully fabricated using the unidirectional solidification method, demonstrating an order of magnitude higher coupling compared to single-crystal Cr_2_O_3_.^12^

Despite the significant technical progress achieved in the development of ME composites, the complexity of their fabrication impeded their practical implementation beyond academic studies. However, beginning in 1990, more simplified and cost-effective fabrication techniques, such as sintering, thin film growth, and epoxy bonding, created renewed interest in the field.^13^ These techniques have enabled the design of various composites, including particulate composites, laminated composites, nanostructured composites, and epitaxial thin films.^14^ At room temperature, the direct ME coupling coefficient for composites ranges from 100 to 400000 mV cm^–1^ Oe^–1^ and can be enhanced at the electromechanical resonance frequency of the structure.^15^ Said differently, the induced voltage is maximized when the applied magnetic field frequency matches the electromechanical resonance frequency of the structure. This frequency depends on the material properties of the transducer as well as its geometry and can range from GHz for nanoscale transducers to a few kHz for cm ones.

While research is ongoing to synthesize single-phase materials with stronger coupling at room temperature,^16−18^ composite materials have already demonstrated their practicality across various applications, including memory devices, sensors, spintronics, and implantable bioelectronics.^2−6,19−23^

**Figure 2:**
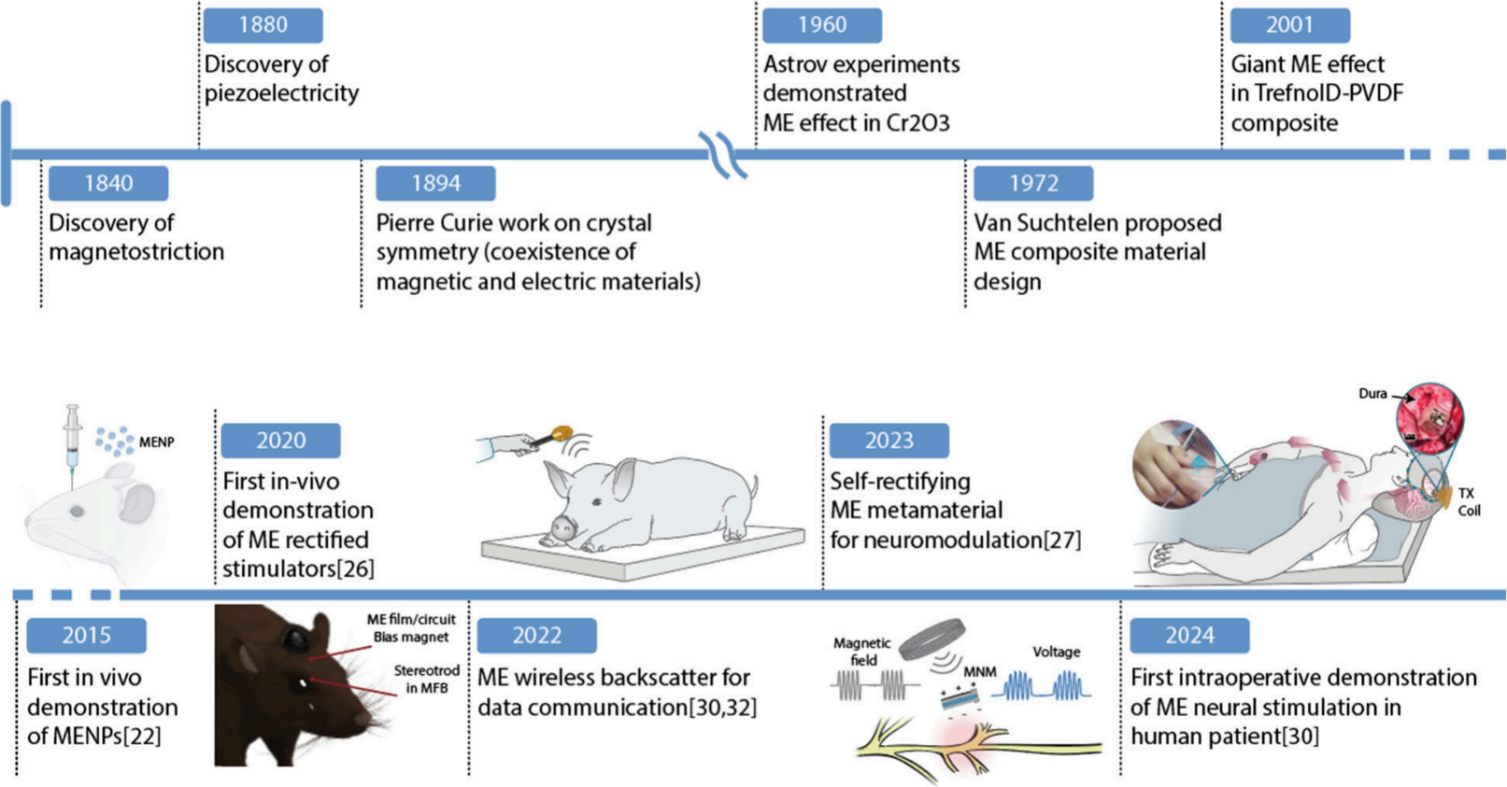
Timeline of magnetoelectric materials history and applications in bioelectronics. Created with BioRender.com. Reproduced with permission from ref (1). Copyright 2020 Elsevier. Reproduced with permission from ref (4). Copyright 2023 Springer Nature. Reproduced from ref (5). Available under a CC BY 4.0 license. Copyright 2024 The Authors.

In the case of implantable bioelectronics, there is a need to create miniaturized solutions that can be delivered with minimally invasive procedures yet have sufficient power supplies and reliable communication systems to ensure stable operation over the therapy period. Over the past decade, there has been an increasing interest in leveraging magnetoelectric materials in this field (Figure2). With high coupling coefficients, these materials can convert the magnetic fields into electric fields that can be used to directly modulate neural activity or transmit data and power to miniature implants.

In this manuscript, we first discuss how ME materials can be used to activate neural tissue directly and then how these materials can be used as part of digitally programmable neural stimulators where the ME material is used to transmit power and data wirelessly. Throughout these sections, we describe proof-of-principle applications where ME-enabled devices and materials have supported in vivo bioelectronics.

## Direct Neuromodulation with Magnetoelectric Materials

One way to exploit magnetoelectric materials in biology is to use the electric field produced by materials in a magnetic field to activate electrically excitable tissue directly. Because electrically excitable cells have voltage-gated ion channels, the electric field produced by a ME material might be able to activate these channels when the material is subjected to a magnetic field. For the ME electric field to be effective in stimulating these tissues, it has to 1) generate an electric voltage greater than the stimulation threshold, which is typically around 15 mV,^24^ and 2) oscillate at frequencies less than 1 kHz to match the slow dynamics of neurons.^25^ However, miniaturized ME transducers generate the highest electric fields around their resonance frequency, which is typically greater than 100 kHz. This is significantly faster than the strength-duration time constants of excitable cells, which range from 20 to 500 μs.^26,27^ When an electric field switches much faster than the membrane time constant, the cell’s membrane potential cannot follow the applied field due to the low pass filtering properties. As a result, high-frequency electrical stimulation is generally ineffective at stimulating action potentials.

**Figure 3:**
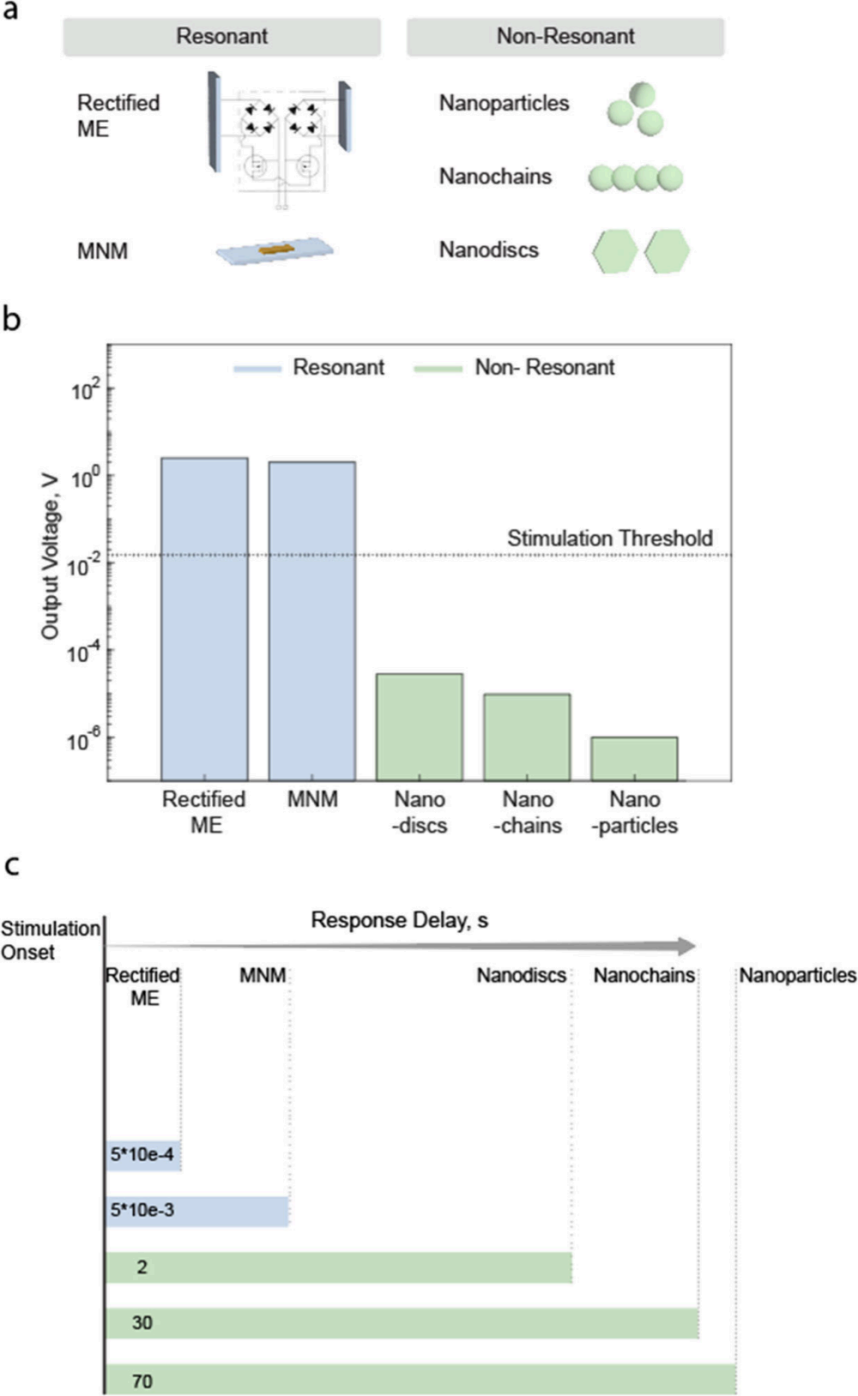
(a) Schematic of resonant and nonresonant strategies and (b) output voltage and (c) response latency measured from the stimulation onset for magnetoelectric off-resonance stimulators (nanoparticles, nanodiscs, and nanochains) and resonance stimulators (rectified ME stimulators and MNM).

There are two potential solutions to enable direct neuromodulation using ME materials (Figure3): 1) operate off-resonance, i.e., drive the ME materials at frequencies low enough for cells to respond, or 2) rectify the electric field produced by the ME material so that the envelope of the electrical stimulation can activate cell activity.

## Off-Resonant Neuromodulation with Magnetoelectric Nanoparticles

Magnetoelectric nanoparticles (MENPs), typically within 30 and 300 nm in diameter, can be delivered to specific regions through intravenous or targeted injection, enabling noninvasive neuromodulation. However, due to their small size, MENPs exhibit GHz resonance frequency, which is significantly faster than the neuronal strength-duration time constant.^28^ To overcome this challenge, MENPs are often excited with off-resonance magnetic fields under 1 kHz. However, at these frequencies, MENPs only generate microvolt voltage stimulation, which is much lower than the neuronal stimulation thresholds. While these voltages are theoretically not strong enough to modulate neural activities, in vivo tests have shown that these particles can still elicit neural responses under specific magnetic field conditions. This phenomenon has been explained through the repetitive subthreshold theory.^29,30^ According to this theory, the repetitive application of magnetic field pulses can influence cell activity. The duration and number of pulses depend on the magnitude of the voltage generated by the MENPs (which is a function of the strength of the applied magnetic field).^31^

The first animal study to investigate the efficacy of MENPs for remote neural stimulation was conducted in 2015.^32^ MENPs were synthesized using a core–shell structure of CoFe_2_O_4_ (magnetostrictive)– BaTiO_3_(piezoelectric) to achieve high ME coupling at the nanoscale. The MENPs were also functionalized with glycerol mono-oleate GMO to eliminate toxicity. These particles exhibit an ME voltage coefficient of 1–100 mV cm^–1^ Oe^–1^, depending on the transition metal used for magnetostrictive core synthesis. MENPs were administered via the tail vein of a mouse model, and a magnetic DC field with a gradient of 3000 Oe/cm was applied to facilitate their crossing of the blood–brain barrier and delivery to the brain. Electroencephalography (EEG) electrodes recorded alternating electric fields at the frequency of the applied magnetic fields (0–20 Hz), but these studies lacked the controls to determine if the EEG signal corresponded to brain activity entrained to the magnetic field frequency or simply the direct ME response of the injected MENPs.

A subsequent study verified that MENPs driven off-resonance could indeed modulate neural activities and produce behavioral changes in mice with more comprehensive control studies to ensure that applied magnetic fields did not entrain these behaviors.^33^ To test that, the CoFe_2_O_4_–BaTiO_3_ nanoparticles were implanted into the subthalamic region of a mouse via stereotactic infusion. An external magnetic field at 140-Hz AC was applied to match the therapeutic frequency for deep brain stimulation to modulate neural activity in the motor cortex and nonmotor thalamus in a freely behaving mouse. The CatWalk behavioral test showed that the MENP stimulation resulted in a significant change in the gait speed. However, these responses were delayed from the onset of stimulation. While the delay was not specified for the in vivo experiment, the in vitro data showed that with the magnetic field of 6 mT applied for 20 s, the change in the Ca^2+^ activity was observed after around 70 s from the stimulation onset. This latency is due to the weak response of the off-resonance MENPs, as explained earlier.

Improving MENPs coupling, hence the generated electric fields could potentially result in stronger and faster responses. Two recent designs have been suggested to engineer MENPs with higher coupling: 1D nanochains and core-double shell nanodiscs.

For 1D nanochains, Fe_3_O_4_@BaTiO_3_ nanoparticles were used to fabricate a 2 μm long nanochain with a height of ∼270 nm.^34^ The enhanced contact area and shape anisotropy resulted in an improved ME voltage coefficient of 2.5 × 10^6^ mV cm^–1^ Oe^–1^, which is ten times higher than the value reported for the Fe_3_O_4_@BaTiO_3_ nanoparticles. It is worth mentioning that while this coefficient is more than 10 × 10^5^ higher than what has been reported for the CoFe_2_O_4_–BaTiO_3_ nanoparticles, the output voltage improvement was less than 10-fold. The nanochains’ in vivo efficacy was tested in an epileptic mouse model to modulate neural activities and suppress seizures. The nano chains were injected into a pilocarpine-induced rat’s anterior nucleus of the thalamus (ANT). After 30 days of implantation, the brain tissue section showed that the nano chains remained stable at the injection site. After inducing a seizure in the animal’s brain, it was exposed to magnetic stimulation at 15 mT at 100 Hz to validate whether the stimulation would prevent seizures from propagating throughout the brain. After 15 min stimulation, the electroencephalogram ECoG signal was suppressed, the seizure duration and stage were reduced, and the animal survival rate improved, confirming the antiepileptic effect of the stimulation. Moreover, the impact of the nano chains was compared to the nanoparticles using the same procedure described above. While both have shown positive results in suppressing the seizure, the nano chains outperform the nanoparticles due to the enhanced magnetoelectric effect.

Disc-shaped nanoparticles with core-double shell synthesized using Fe_3_O_4_–CoFe_2_O_4_–BaTiO_3_ have shown an ME coupling coefficient of 15 mV Oe^–1^ cm^–1^, which is four times higher than that of spherical CoFe_2_O_4_–BaTiO_3_ particles.^31^ The improved ME coupling observed in these nanodiscs can be attributed to two key design choices. First, disc-shaped nanoparticles were fabricated instead of spherical ones, which resulted in an increase in the shape anisotropy and magnetocrystalline anisotropy, leading to higher magnetostriction. Second, the magnetostrictive layer comprised two different materials with varying magnetostriction constants, which enhances the generated strain.

At an applied magnetic field of 5 mT, a 250 nm diameter ME nanodisc generates >24 μV. Kim et al. proposed that according to repetitive threshold theory, these MENPs depolarize the neural membrane potential from −70 to −55 mV when the field is applied for 2 s.^31^

To evaluate the efficacy of this design in vivo, a low dosage of 1.5 μL (1.5 mg/mL) solution (compared to 2 μL 100 mg/mL for nanochains) of the ME nanodiscs is injected into a mouse brain’s left ventral tegmental area (VTA). Applying an external magnetic field at 150 Hz substantially increased C-fos expression and GCaMP6s fluorescence upon imaging. Subsequently, the mouse was subjected to a custom three-chamber assay to evaluate whether ME stimulation could induce a place preference, a standard experiment to investigate dopamine-dependent reward circuits. The experimental results showed that the animals preferred the stimulation chamber, thereby confirming the successful stimulation of VTA using ME nanodiscs.

Despite the promising avenue for remote neural modulation in the studies above, MENPs have limitations due to the slow biological response when the particles are driven off-resonance. Because the field is so weak, they have relatively low temporal resolution with latencies ranging from 100 ms to seconds. The mechanism of neuronal modulation using these particles needs further investigation to enhance their responsiveness and expand their application beyond deep brain stimulation to applications that require timely response, such as neuroprosthetics. To improve the efficacy of MENPs, a comprehensive study of various parameters that may affect the stimulation is necessary. This includes nanoparticle size, concentration, and applied magnetic field parameters. It is worth noting that none of the studies conducted so far report controllability over the stimulation parameters, such as the stimulation amplitude, and whether it can be linearly controlled by modulating the applied field intensity. Furthermore, examining these particles’ lifespan, functionality, efficacy, and immune response over time is crucial. Animal model testing must also be expanded beyond small animal models to provide more translatable results in case of clinical applications.

## Resonant Neuromodulation with Rectifying Magnetoelectric Stimulators

To address the issue of slow and weak biological response resulting from off-resonance operation, we have developed ME stimulators that can be excited with high-frequency magnetic fields that match the mechanical resonance of the material but can rectify the generated electric field, resulting in a low-frequency envelope that can efficiently activate voltage-gated ion channels. We have demonstrated two different ways to rectify the magnetoelectric effect: 1) using external analog components or 2) engineering self-rectifying ME materials. In both designs, we use ME laminates consisting of a Metglas layer (magnetostrictive material) and a PZT-5 layer (piezoelectric material) to create the ME transducer. We attach a sheet of Metglas and PZT-5 using a thin layer of epoxy and then use a laser cutter to obtain an ME transducer with mm-size. At this scale, the ME transducers have resonance frequencies of 100–500 kHz.^35^ A list of other ME composites that have been used for biomedical applications can be found in.^36^

The first design for the resonant ME stimulator is an analog device that combines two ME transducers and basic electronic components such as diodes, switches, and LEDs.^1^ We designed the ME transducers to have slightly different lengths so that we could selectively activate them by changing the magnetic field frequency. Using two ME films was essential for generating biphasic stimulation, a common practice to ensure electrode safety and charge balance. Moreover, we designed a custom magnetic field transmitter to control the amplitude and frequency of the applied magnetic field, hence the delivered stimulation. The ME transducers operate at 180 and 200 kHz frequencies and generate voltages exceeding 5 V. However, we limit the output voltage to 1.8 V using Zener diodes or LEDs for safety. Our in vitro testing showed that we can successfully elicit action potentials synchronized with the applied magnetic field with less than 1 ms latencies. Moreover, we used the device to remotely stimulate deep brain regions in freely moving hemiparkinsonian rats. The device delivers ±1.5 V, which corresponds to approximately ±100 μA stimulation current based on the average measured impedance values of the electrodes, which were 10k ohm for each electrode. During a behavioral test, we observed a change in angular velocity when the applied field is pulsed at 200 Hz, the therapeutic frequency for deep brain stimulation. However, a major limitation of this design is miniaturization; while the size of the ME transducers used here was 2–4 mm^3^, the packaged implant was 175 mm^3^ due to discrete analog components.

To create even smaller solutions with a rectified magnetoelectric effect, we created magnetoelectric nonlinear metamaterials (MNM) that exhibit self-rectification characteristics. When excited by high-frequency magnetic fields, this new material generates a rectified wave whose envelope can activate neural responses with 5 ms latency.^4^

The monolithic structure of the MNM allows for more miniaturized stimulators compared to analog circuits. In this case, the MNM is fabricated using a ME laminate and a rectifying electron transport layer (RET). Specifically, we fabricated a trilayer ME laminate of Metglas-PZT-5-Metglas, then deposited a thin layer of 130 nm of ZnO and 40 nm of HfO_2_ to create a nanoscale RET to produce the rectification effect. The RET layer behaves like a diode, so the output electric field is no longer a linear function of the magnetic field, as in the case of the ME transducer. Instead, the MNM behavior can be more accurately described by a third-order polynomial function as follows: *V* = α_1_*H* + α_2_*H*^2^ + α_3_*H*^3^.

Due to this nonlinearity, a bias voltage is developed across the MNM when excited by a high-frequency resonance magnetic field. We have demonstrated that a 5 × 3 mm^2^ MNM can generate a 1.7 bias voltage when excited with a 2 mT magnetic field at 335 kHz. To verify that this voltage level is sufficient to elicit sensory neural responses, we have placed MNM directly on the sciatic nerve of an anesthetized rat model and delivered precise neural stimulation that induced leg kicks and EMG activities. Furthermore, we have utilized the fast response of the MNM to develop a neuroprosthetic platform that can restore nerve conduction across a severed rat sciatic nerve. The MNM delivers electrical stimulation to activate the distal muscle groups of the animal with latencies of less than 5 ms. This represents a 120-fold improvement in latency over any previously reported remote neural stimulation using magnetic materials.

For both designs above, we tune the stimulation amplitude by changing the applied field strength, which may lead to changes in output stimulation if the transmitter and implant are misaligned. However, for clinical applications, we must precisely control stimulation to ensure safety and efficacy.

## Programmable Magnetoelectric Stimulators

**Figure 4:**
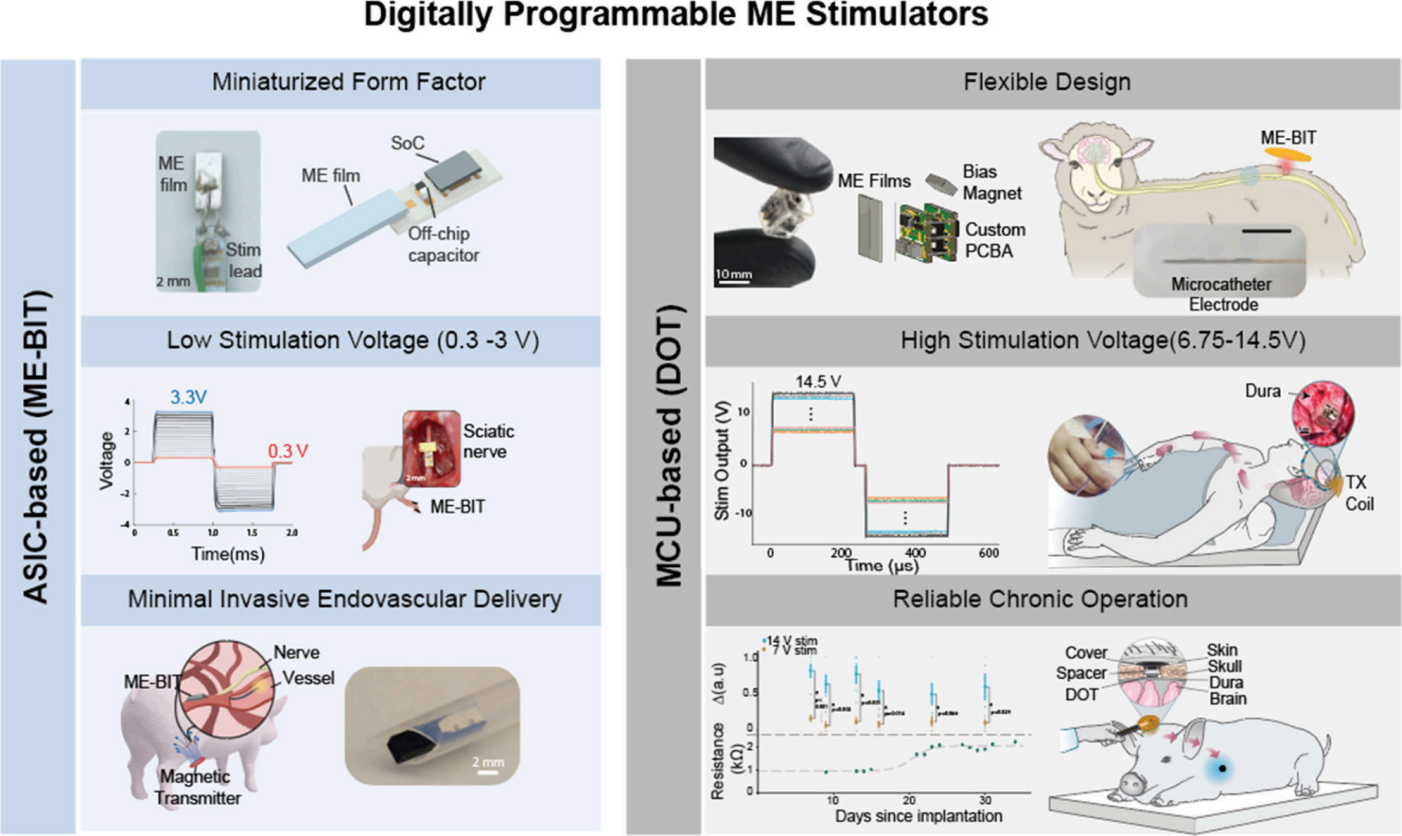
Digitally programmable ME stimulators: ASIC-based (ME-BIT)^2^ and MCU-based (DOT).^5,39^ Reproduced from ref (2). Available under a CC BY 4.0 license. Copyright 2022 The Authors. Reproduced from ref (5). Available under a CC BY 4.0 license. Copyright 2024 The Authors. Reproduced from ref (39). Available under a CC-BY-NC-ND 4.0 license Copyright 2024 The Authors.

To achieve the precision and control required for biomedical devices, we have recently created digitally programmable stimulators that are powered by ME materials (Figure4). These devices rely on magnetic-to-electric field conversion as an efficient wireless power *and* data transfer method and expand the types of electrical waveforms that can be delivered to the tissue. The devices are programmed by modulating the applied magnetic field, which is then demodulated into a digital bit sequence that encodes the programming data. By digitally programming devices, we can better ensure that they provide safe and effective stimulation even if the coupling between the transmitter and receiver fluctuates.

As a data and power transceiver for these implants, we optimized the ME laminates consisting of the same Metglas-PZT5 reported in the previous sections to enhance the power transfer efficiency. We have shown that optimizing the design parameters, particularly thickness ratio and mechanical coupling, can get an ME coupling factor of 2 V/Oe and a power density of 3.1 mW/mm^2^.^37^ For instance, a 9 × 3 mm^2^ film can receive a maximum power of 56 mW, which is sufficient to power electronic components and deliver electric stimulation for various neuromodulation applications. The features and limitations of this approach compared to other modalities, including electromagnetic, photonic, and acoustic techniques, can be found in.^38^

Based on these transceivers, we created MagnetoElectric BioImplanT (ME-BIT), the first programmable ME neural stimulator that integrates the ME technology with the complementary metal-oxide-semiconductor (CMOS) technology. Specifically, the device combines an ME transducer with an application-specific integrated circuit (ASIC) designed using the 180 nm CMOS technology. The ASIC is only 1 × 0.8 mm^2^ in size, consumes a few microwatts of power, and offers multiple functions, including power management and controlled stimulation.

A key advantage of this design is miniaturization; the ME-BIT device has a footprint of 6.2 mm^3^, which is more than ten times smaller than the analog version and allows wireless power and digital data downlink through the ME link. The wireless downlink enables the control of neural stimulation parameters like frequency, shape, duty cycle, and amplitude. To enable this feature, we exploit the ME output dependence on the applied field frequency; when the applied field frequency is detuned from the resonance frequency, there is a significant drop in the ME film output voltage. Hence, the frequency modulation of the transmitter field results in amplitude modulation of the ME film voltage. In the case of ME-BIT, the device can be remotely controlled to deliver stimulation voltage with an amplitude ranging from 0.3 to 3.3 V, pulse width from 0.05 to 1.2 ms, and delay from 0.01 to 0.8 ms.^2^

For a proof of concept of the device operation in vivo, we have shown that ME-BIT can deliver effective electric stimulation to excite peripheral nerves in rodent and porcine models. To demonstrate the stimulation controllability, we placed ME-BIT on the sciatic nerve of a rat model and controlled the amplitude of the delivered stimulation. Once the stimulation was delivered, we recorded muscle action potentials (CMAPs) synchronized with leg kicks. Increasing the stimulation amplitude was associated with increased response.

Leveraging the device’s miniaturized form factor, we showed that ME-BIT could be percutaneously deployed through a 9 Fr catheter into the femoral artery of a pig model. This approach enabled endovascular stimulation of the femoral nerve through the femoral artery at various frequencies. We also demonstrated the ability to stimulate the intercostal nerves and the dorsal root ganglion, typical targets for treating chronic pain entirely with minimally invasive procedures.

While the ME-BIT voltage compliance of a maximum of 3.3 V is sufficient for many peripheral nerve targets, it cannot support applications that require higher stimulation thresholds, like epidural cortical stimulation. This limitation resulted not from the power capabilities of the ME transducers but rather from the low-voltage compliance of the CMOS technology used to make these ASICS.

To overcome this limitation, we developed a Digitally programmable Overbrain Therapeutic (DOT) that can deliver high stimulation levels of up to 14.5 V.^5^ To achieve this high-voltage compliance, we combined our ME transducer with a custom-designed printed circuit board and off-the-shelf components with a microcontroller unit (MCU), taking the place of a custom ASIC. The DOT can be programmed to output 250 and 500 μs pulse width biphasic stimulation pulses with 6.75–14.5 V amplitude in 250 mV increments. Based on an estimated electrode impedance of 1k ohm, the stimulation current ranges from 6.75 to 14.5 mA. The ME transducer is 7 × 3.5 mm^2^, whereas the fully packaged device is 11× 9 × 9 mm^3^. To validate this design’s efficacy, we tested the device intraoperatively during a neurosurgical procedure for two human patients. The DOT was placed directly on the motor cortex; hence, when activated, it stimulated a motor response captured as hand contraction. To confirm that our device could provide this level of stimulation over time, we used a porcine model to test the chronic operation of the device. The DOT could drive motor stimulation consistently when implanted in pigs for 20–35 days above the dura over the motor cortex.

The DOT’s flexible design enables the integration of different electrode configurations to design customizable neural interfaces. We have shown that by integrating the DOT with flexible catheter electrodes, we can create an endocisternal neural interface (ECI) that can be used to approach brain and spinal cord targets through inner and outer cerebral spinal fluid (CSF) spaces in a sheep model.^39^ We demonstrate multisite electrical stimulation and recording through the ECI at the motor cortex, deep brain structures, and throughout the spinal cord from lumbar to cervical regions.

To generate sufficient magnetic fields to power the different ME devices listed in this section, we have built battery-powered custom wearable transmitters, hence eliminating the need for direct wire connections to external power supplies.^35^ The system combines power electronics and a resonance coil tuned to match the resonance frequency of the ME transducer. By carefully designing the coil, we can generate a sufficiently strong magnetic field at specific depths within biological tissue. Furthermore, using a single transmitter coil, we can simultaneously power a network of ME stimulators for coordinated multisite stimulation. The network can be scaled to multiple devices without increasing the transmitter power, as these devices do not load the transmitter coil.^40^ To create fully wireless systems for animals, it should be possible to generate magnetic fields in arenas where animals can be free to behave similarly to cages used for RF power delivery.^41^ In all cases, it is crucial to ensure that the generated magnetic field remains within established safety limits for in vivo applications.

## Wireless Communication

Integrating ME devices with sensors would advance the device’s capabilities to provide diagnosis and deliver adaptive therapeutic interventions; however, establishing an uplink communication system that can transmit the sensor data from the implant to external transceivers is a major limitation. An ideal solution would be to use the same ME link for wireless power and bidirectional communication to maintain the miniaturized device form factor and simplify the overall design.

Toward this goal, we have developed the first passive ME communication system, leveraging two fundamental characteristics of the ME transducer. First, ME materials generate a backscattered magnetic field when excited by an external field; we exploit these fields as a carrier signal. Second, the characteristics of the backscattered field can be modulated by an external electric load; thus, we can use load modulation for digital data encoding.

These unique characteristics are mainly inherited from the magnetostrictive and piezoelectric layers, respectively. When subjected to an external magnetic field, magnetostrictive layers generate mechanical vibrations due to the Joule effect. These vibrations lead to a change in the material magnetization that appears as a backscattered magnetic field generated by the material. The caveat to using this backscattered field as a carrier signal is that it oscillates at the excitation field frequency; hence, decoupling it from the excitation field is extremely challenging. To avoid this problem, we measure the backscattered field during the ring-down period when the excitation field is turned off. To encode digital data, we modulate the characteristics of the backscattered field, such as amplitude and frequency, exploiting the piezoelectric material’s tunability using an external electric load. Specifically, loading the piezoelectric laminate with an electric load modulates the effective mechanical properties, which, due to the mechanical coupling between the piezoelectric and magnetostrictive layers, in turn, modulates the effective mechanical properties of the ME transducer, hence modulating the backscattered field characteristics. We typically rely on resistive or capacitive loads to avoid electromagnetic interference issues.

**Figure 5:**
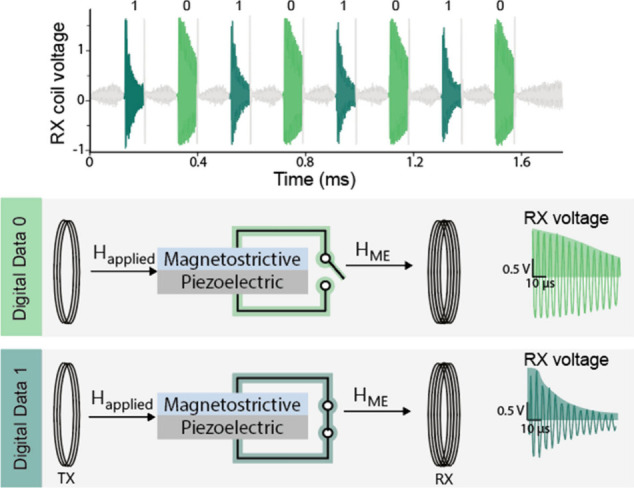
Illustration of digital data transmission using ME backscatter communication.

The primary advantages of this design are that it needs an ultralow-power budget and it maintains the miniaturized device size. The only components required to design the uplink communication module are a passive load and a switch, both of which are at least an order of magnitude smaller than the ME transducer. As a proof of concept, we have implemented this design using a 1 × 1.5 mm^2^ ASIC chip combined with a 2 × 5 × 0.28 mm^3^ ME transducer. The ASIC chip switches a capacitive load connected across the ME transducer to modulate the frequency of the backscattered field. To detect this field outside the body, we designed a custom transceiver (TRX) that combines a sensitive pickup coil and a magnetic transmitter. The system achieves a data rate of 8 kbps with a bit error rate of less than 1 × 10^–3^ when operated at a carrier frequency of 335 kHz and distance of more than 20 mm. To demonstrate the system performance in vivo, we combined the DOT device with an uplink module that switches between open circuit and short circuit conditions to modulate the amplitude of the backscattered field (Figure5). While implanted over the dura of a pig model, the device successfully communicated the device electrode’s impedance over an implantation period of 30 days.

## Conclusion, Outlook, Discussion, and Perspective

Throughout this Account, we have demonstrated the potential of ME materials to create miniaturized bioelectronic solutions for various neuromodulation applications. These materials can be used to modulate neural activities directly, wirelessly receive power and data for digitally programmable stimulation, or wirelessly transmit data for remote monitoring.

Despite the considerable progress in the field, there are still many opportunities to leverage the ME technology to develop next-generation bioelectronics that feature networks of distributed implants and adaptive systems, enabling more personalized therapeutic interventions and precise diagnostics.

One could further miniaturize the device size to the micrometer scale by engineering magnetoelectric materials with higher ME coupling, enabling minimally invasive technologies like injectable or ingestible devices. Moreover, enhancing the material’s biocompatibility and flexibility would allow seamless integration with tissue and longer stable operation.

To expand the stimulation modalities enabled by this technology beyond electrical stimulation, these materials can be combined with other smart materials to design new composites that could generate optical, thermal, or mechanical stimulation in response to magnetic fields that have excellent tissue penetration abilities.
